# Adipogenicity-induced human mesenchymal stem cells treated with hemp seed oil stimulate brown-like adipocytes and decrease adipokine levels through the activation of cannabinoid receptor 2 (CB2)

**DOI:** 10.1186/s42238-025-00343-2

**Published:** 2025-11-19

**Authors:** Albatul Almousa, Pandurangan Subash-Babu, Ibrahim O. Alanazi, Ali A. Alshatwi

**Affiliations:** 1 Department of Food Science and Nutrition, College of Food and Agriculture Sciences, King Saud University, Riyadh, 11451 Saudi Arabia; 2 Genome Research Chair, Department of Biochemistry, College of Science, King Saud University, Riyadh, 11451 Saudi Arabia

**Keywords:** Stem cells, Hemp seed oil, Cannabinoid receptors, Adipogenesis, Brown-like adipocytes, Inflammation

## Abstract

The endocannabinoid system (ECS) is essential for energy hemostasis, obesity, and other metabolic disorders. Cannabidiol and polyunsaturated fatty acids (PUFAs), which are found in hemp seed oil (HSO), have been found to regulate adipose tissue through the ECS. Thus, human mesenchymal stem cells (hMSCs) were differentiated into pre-adipocytes and then treated with cannabidiol (CBD), tetrahydrocannabinol (THC), 0.05% HSO, or 0.1% HSO for 3 days (72 h). The mixture was subsequently maintained in maintenance media for 14 days, after which the condition media (CM) was collected. In addition, THP-1 cells were used to assess the inflammatory response upon exposure to CM collected from different groups of experimental cells. Quantification for lipid accumulation (Oil red O), gene expression (RT‒qPCR), and protein levels (Western blot) were performed. We found that HSO-treated cells matured toward brown-like adipose tissue with a spindle shape and decreased intracellular lipid accumulation. HSO treatment decreased the expression of genes associated with fat accumulation and browning (BAT), with the exception of UCP-1, which leans toward brown-like adipocytes. HSO treatment upregulated the cannabinoid receptors 2 (CB2), TRPV1, and GPCR55 mRNAs and leptin mRNA found with lower expression; no alterations were observed in cannabinoid receptors 1 (CB1), FAAH, and MGL mRNAs. In THP-1 macrophage, HSO treated CM decreased the expression of IL-6, IL-8, TNF-α, and leptin mRNAs significantly when compared to CBD and THC. The potential of HSO in promoting brown fat characteristics through the CB2 and its effect on inflammation status offers an intriguing area for future research and therapeutic interventions.

**Supplementary Information:**

The online version contains supplementary material available at 10.1186/s42238-025-00343-2.

## Introduction

Hemp seeds from nondrug cultivars of *Cannabis sativa* L. have been extensively utilized for several years as valuable sources of medicines, edibles, and textiles (Claro-Cala et al. 2022). Hemp seed oil (HSO) comprises cannabinoids such as cannabidiol (CBD) and polyunsaturated fatty acids (PUFAs), including balanced ratios of omega-3 and omega-6 (Farinon et al. 2020). CBD and PUFA (omega-3) have been found to regulate adipose tissue through different mechanisms. Research has shown that dietary lipids have distinct effects on gene expression in white and brown adipose tissues. Specifically, the consumption of the dietary PUFA omega-3 increases uncoupling protein 1 (UCP 1) mRNA levels in brown adipose tissue, potentially leading to a decrease in body fat accumulation (Takahashi and Ide 2000). Furthermore, CBD has been found to stimulate fat cell formation in MSCs through peroxisome proliferator-activated receptor gamma (PPARγ) (Chang et al. 2022). Additionally, the use of CBD treatment has the potential to effectively stimulate the brown adipose tissue characteristics in white adipocytes by significantly increasing the expression of genes and proteins specific to thermogenesis (Parray and Yun 2016). In addition, CBD and omega-3 PUFAs influence the levels of endocannabinoids in adipose tissue (Batetta et al. 2009; Chang et al. 2022). Endocannabinoids are part of the endocannabinoid system (ECS), which is vital for energy hemostasis. The potential of HSO to promote the development of brown adipocyte characteristics through the ECS offers an intriguing area for future research and therapeutic interventions.

In addition to its role in controlling vital physiological processes, such as sleep, hunger, pain, inflammation, memory, and reproduction (Kuzumi et al. 2024), the ECS controls the metabolism of adipose tissue, and the activation of this system leads to obesity and metabolic disorders (Spoto et al. 2006; van Eenige et al. 2018). The ECS maintains its balance through the cellular endocannabinoids anandamide (AEA) and 2- arachidonoyl glycerol (2-AG). These cellular endocannabinoids bind to specific cannabinoid receptors 1 and 2 (CB1 and CB2) and noncannabinoid receptors such as, G protein coupled receptor 55 (GPCR55) and transient receptor potential vanilloid 1 (TRPV1) (Ryberg et al. 2007; Spoto et al. 2006). Since the discovery of CB1 (Bensaid et al. 2003; Cota et al. 2003) and CB2 (Roche et al. 2006) from adipocytes, the research focus on the exploration of its functional role on lipid metabolism. Research has shown that signaling through CB1 increases lipogenesis (Simon and Cota 2017) and hinders the formation of new mitochondria (Tedesco et al. 2010). In contrast, the activation of CB2 stimulates the conversion of adipocytes into brown fat cells (Rossi et al. 2016). In addition, fatty acid amyl glycerol (FAAH) and monoacyl glycerol (MGL) are ECS enzymes that breakdown endocannabinoids. Moreover, diacylglycerol (DAGL) and phospholipase A1 (PLA1) are enzymes needed for the synthesis of endocannabinoids (Bie et al. 2019).

Adipose tissue serves both as a reservoir for storing extra energy and as a metabolic endocrine organ that releases adipokines, cytokines, and growth factors (Fellous et al. 2020). These substances work together to regulate the overall balance of energy and inflammation in the body. There is growing evidence that the ECS plays a role in maintaining these substances, leading to healthy adipose tissue (Komarnytsky et al. 2021; van Eenige et al. 2018). Furthermore, CBD and omega-3 PUFAs may exert their effects as anti-inflammatory substances through the ECS (Brown et al. 2013; McDougle et al. 2017). Both compounds are present in HSO, which has been found to exert an anti-neuroinflammatory effect on BV2 microglia after inflammation is induced by lipopolysaccharide (Zhou et al. 2018). The potential of HSO to reduce inflammation through the ECS offers a promising direction for future research and therapeutic applications.

The chemical composition of HSOs might represent different aspects of the involvement of the ECS in adipose tissue and inflammation. Nevertheless, as all types of adipocytes originate from MSCs, we must consider them therapeutic targets for understanding the regeneration of functional adipose tissue and the re-establishment of energy metabolism (Lizcano 2019; van Eenige et al. 2018). Hence, our previous research revealed that HSO initiated adipogenic differentiation in hMSCs without differentiation agents and regulated the ECS by suppressing CB1 gene and protein expression (Almousa et al. 2024). Thus, this research aimed to evaluate the involvement of the ECS in the maturation of hMSC preadipocytes to mature adipocytes and its effect on inflammatory markers upon treatment with HSO compared with CBD and THC.

## Materials and methods

### Products and chemicals

The trials employed cold-pressed HSO from iHerb in two doses: 0.05% low dose (0.05% HSO) and 0.1% high dose (0.1% HSO). RESTEK^®^ provided liquid CBD and THC standards. HPLC-grade methanol and isopropanol were gifted from King Abdul-Aziz City for Science and Technology, Riyadh, Saudi Arabia.

### Cell culture

Human mesenchymal stem cells (PCS-500-012), originally was purchased from American Type Culture Collections (ATCC), Manassas, Virginia, USA. Present study, we obtained the hMSCs from Dr. Ali A Alshatwi’ lab, College of food and Agricultural Science, King Saud University’s as generous gift. The cryopreserved cells were thawed (passage 1) and immediately transferred to culture flask containing DMEM (Gibco, Waltham, MA, USA) supplemented with 10% FBS (FBS, Gibco™, NY, USA) and 1% penicillin‒streptomycin (Gibco, NY, USA). The cells were maintained at 37 °C in a 5% CO_2_ humidified atmosphere until 70% confluency. Afterward, the cells (≤ passage 2) were grown in differentiation media (DM) consisting of dexamethasone (400 ng/ml), IBMX (44 µg/ml), Troglitazone (3 µg/ml), and insulin (0.5 µg/ml) for 72 h (PromoCell, 2021). In the control group, adipogenic differentiated hMSCs (D-hMSCs) were cultured in maintenance media (MM) [Complete growth media added with 10 µg/ml of insulin] for 14 days, and the media was changed every three days. For the experimental groups, 1 µM CBD, 1 µM THC, 0.05% HSO, or 0.1% HSO was added for 72 h. HSO was emulsified with 1% Tween-20 for homogenization, while CBD and THC were dissolved in methanol. For the final seven days, the cells were maintained in MM, and the media was not changed until the end of experiment. The experimental cell secreted adipokines or microparticles containing conditioned media (CM) were collected without disturbing the adherent adipocytes to treat THP-1 cells.

THP-1 cells were a gift from Dr. Yasser Ba-Ismaeel, a professor at the College of Science and Health Professions at King Saud bin Abdulaziz University for Health Sciences. The cells were cultured in RPMI 1640 media supplemented with 20% FBS and 1% penicillin‒streptomycin at 37 °C in a humidified atmosphere of 5% CO_2_ until their viability reached 95%; then, the cells were subcultured into six groups and incubated until they reached 70% confluency. The media was then replaced with CM collected from the previous experiment. After 2 h of incubation with CM, the cells were exposed to inflammation caused by the addition of 100 ng/ml lipopolysaccharide (LPS) and incubated for 24 h. The groups were as follows: negative control (maintenance media only), positive control (+ LPS), control (D-hMSC-conditioned media), CBD (AD-CBD-conditioned media), THC (AD-THC-conditioned media), and 0.1% HSO (AD-0.1% HSO-conditioned media).

### Oil red O & nile red staining

A 6-well plate was seeded with hMSCs and allowed to reach 70% confluency. The cells were treated with DM for 72 h and then with CBD, THC, 0.05% HSO, or 0.1% HSO for 72 h. The maintenance media was subsequently changed every three days until day 7, final 7 days kept the cells as undisturbed. On day 14, the cells were rinsed with PBS, fixed with 10% isopropanol at room temperature for 1 h, and washed three times with deionized water. The oil red O (ORO) solution (6%) dye was dissolved in 6:4 isopropanol in water, applied to the cells for 10 min, and then washed with deionized water until it became transparent. Nile red (NR) staining requires discarding the medium and washing with PBS, adding a 1:1 combination of NR and deionized water and incubating at room temperature for 10 min before imaging. A ZEISS Axiocam 506 inverted microscope was used to capture the stained lipid droplets. Following overnight removal of ORO bound with 100% isopropanol (400 µl/well), intracellular lipids were quantified via a microplate reader, and 620 nm absorbance was measured in triplicate wells.

### RNA extraction and real-time polymerase chain reaction (RT‒PCR)

The cells were harvested on experiment day 14, and total RNA was extracted via the RNEASY MINI Kit (Qiagen, Inc. 74106, USA). We tested the concentration and purity of the extracted RNA with a QuickDrop instrument (SpectraMax, USA). A high-capacity cDNA reverse transcription kit (Applied Biosystems by Thermo Fisher Scientific, Lithuania) was used to convert 1 µg of RNA to cDNA. QuantStudio 3 (Thermo Fisher Scientific, USA) and SYBR^®^ green Universal Master Mix (Life Technologies, UK) were used to measure gene transcription. For each sample, PCR was repeated twice, and gene transcription was standardized to that of GAPDH. The same technique was used for the inflammatory experiment (THP-1). The list of primers used is presented in Table 1..

**Table 1:** Sequences of the primers used for qRT‒PCR

Primers	Sequence
GAPDH-F	5′-GAGTCCACTGGCGTCTTC-3′
GAPDH-R	5′-GGGGTGCTAAGCAGTTGGT-3′
PPARγ-F	5′-GACCAGAAGCCTGCATTTCTGC-3′
PPARγ-R	5′-CTGTGTCAACCATGGTCATTTCGTT-3′
CEBPα-F	5′-AGGAGGATGAAGCCAAGCAGCT-3′
CEBPα-R	5′-AGTGCGCGATCTGGAACTGCAG-3′
FABP4-F	5′-TGACAGGAAAGTCAAGAGCACC-3′
FABP4-R	5′-AACTCTCGTGGAAGTGACGC-3′
PLIN1-F	5′ - GGTCAGCCGGACTTGAGGA-3′
PLIN1-R	5′- TCTGGAAGCATTCGCAGGTG-3′
PPARα-F	5'- GCCTCCTTCGGCGTTCG -3'
PPARα-R	5'- CCGAGCTCCAAGCTACTGTG -3'
PGC1α-F	5′- ATGAAGGGTACTTTTCTGCCCC-3′
PGC1α-R	5′- ATTGCTGATGCTGCTTGCAC-3′
PRDM16-F	5′-GGCAGGCTAAGAACCAGGCAT-3′
PRDM16-R	5′-GAGGGGGTGTGGAGAGGAG -3′
UCP1-F	5′- CTCAGGATCGGCCTCTACGA-3′
UCP1-R	5′- TGCTTCCTAAACTAGGTGCTGTTTC-3′
CB1-F	5′- GTTCTAGCGGACAACCAGCC-3′
CB1-R	5′- TCAATCTCTTTGCCCCTTCGC-3′
CB2-F	5′- ACTCAACAGGTGCTCTGAGTGG-3′
CB2-R	5′- CTTGTCTAGAAGGCTTTGGGTTGTG-3′
TRPV1-F	5′- TTCGAGTAGCAACCGCCTTC-3′
TRPV1-R	5′- CCCAGTGTGCAACCAGCTAGA-3′
GPCR55-F	5′- ATGACATCTCTCAGCCCTCTCAG-3′
GPCR55-R	5′- ATCAGCTCGTTGACACCGTC-3′
FAAH-F	5′- GAGGACATGTTCCGCTTGGA -3′
FAAH-R	5′- AAGAAGGGAACCAGCGTGTG -3′
MGL-F	5′- GCAAACGAGGATCCGCTGC-3′
MGL-R	5′- GGGAGGTCCTGGTAGGGAAT-3′

### Immunoblot analysis

RIPA lysis buffer and Halt™ protease inhibitor (PI) (Thermo Fisher Scientific, USA) were used to generate cell lysates at a 1:25 ratio. After homogenization on ice for an hour, the cell lysates were centrifuged for 30 min at 14,000 rpm. A Bradford test (Bio-Rad protein test kit, USA) was used to measure the protein concentration. All the samples had a total protein concentration of 35 µg/ml. To create the sample buffer, a 1:19 ratio of β-mercaptoethanol to 2x-laemmli-sample buffer was used. The samples were mixed 1:1 with sample buffer and heated at 95 °C for 5 min before being subjected to 10% SDS‒PAGE. After electrophoresis, the proteins were transferred to a 0.2 μm PVDF blotting membrane (Amersham™ Hybond™, Germany) and blocked with 5% skim milk for 1 h. The membrane was washed three times with 15 ml of TBST 1X buffer containing 50 ml TBS 20X + 950 ml DW + 1 ml Tween 20 and then incubated overnight with 1:4000, 1:200, 1:500, and 1:166 dilutions of the primary monoclonal antibodies anti-CB1 (ab259323), anti-CB2 (ab150569), anti-TRPV1 (ab6166), and anti-GPCR55 (ab203663) were obtained from Abcam (Cambridge, United Kingdom). After 2 h of incubation at room temperature with the primary monoclonal antibody anti-GAPDH (1:10,000 dilution), GAPDH (ab9485) was utilized as a housekeeping protein. The membrane was then treated for 1 h with Abcam’s 1:1000 horseradish peroxidase-conjugated anti-(rabbit/mouse) IgG secondary antibody in 5% skim milk. After the membranes were incubated with the Clarity Western ECL Substrate Kit, images were acquired via a ChemiDoc™ Touch Imaging System (Bio-Rad, USA). The band intensities were measured with Image Lab.

### Statistical analysis

The biological experiments data presented as mean and standard deviation (SD) of quadruplicate technical replicates (*n* = 4) have been used to represent all the data. In addition, one-way ANOVA was used to examine the statistical significance among multiple groups, followed by the Dunnett test for multiple comparisons using GraphPad Prism 10.2.3 (Windows, GraphPad Software, San Diego, California USA, www.graphpad.com). GraphPad Prism 10.2.3 program will not calculate/include the standard deviation for untreated control or negative control. Statistical significances were indicated as *p*-values **p ≤ 0.01, ***p ≤ 0.001.

## Results

### HSO decreased the intracellular lipid content and regulated genes related to adipogenesis and brown adipose tissue

**Fig. 1:**
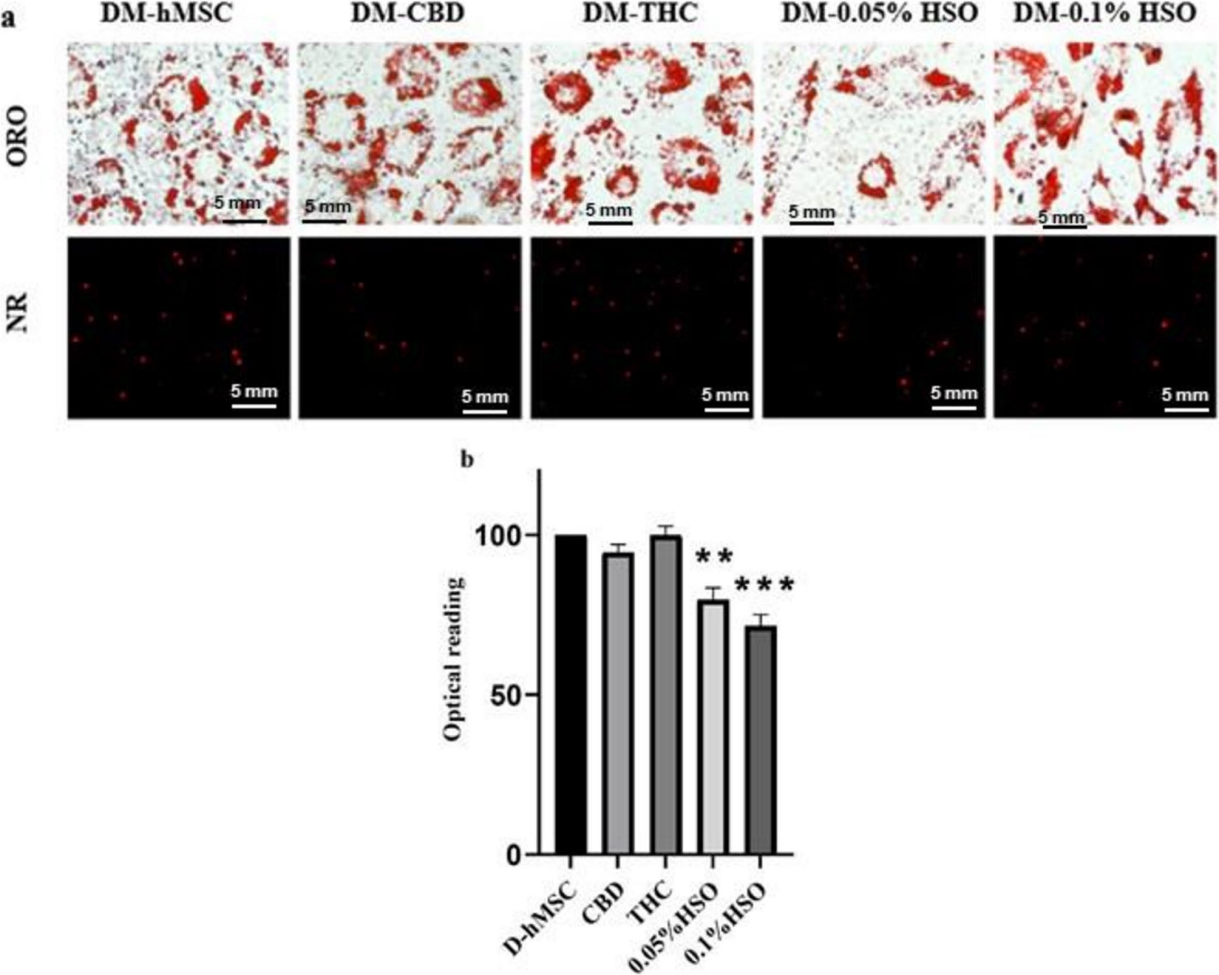
Oil Red O & Nile Red stain and fluorescence dye. **a** hMSC morphological alterations following 72h DM, 72h CBD, THC, 0.05% HSO, or 0.1% HSO, and MM until day 14.**b** ORO density in 100% absolute isopropanol. The biological experiments data presented as mean and standard deviation (SD) of quadruplicate technical replicates (n=4) were used to represent all data (mean ± SD), are shown; ANOVA as ** *P* ≤0.01, *** *P* ≤0.001

We sought to study the morphological changes in differentiated hMSCs and the intracellular lipid content following treatment with HSO, CBD, or THC. To this end, hMSCs were cultured in differentiation media for 72 h, followed by 72 h of treatment with CBD, THC, 0.05% HSO, or 0.1% HSO, and then maintenance media every 3 days until they reached the 14^th^ day. ORO and NR staining confirmed hMSC adipogenic differentiation. Fig. 1(a) shows that cells treated with HSO (0.05% and 0.1%) have a spindle shape rather than the round shape noted in the D-hMSC, CBD, and THC. In the intracellular lipid content quantification test, all the treatment groups presented lower lipid contents than did the control (D-hMSC); however, the HSO-treated groups (0.05% HSO and 0.1% HSO) presented the lowest lipid contents, with values of 20.25% ±3.684 and 28.44% ±3.554, respectively (Fig. 1(b)).

Next, we aimed to study the genomic changes in mature adipocytes upon different treatments. To this end, the gene expression of adipogenic differentiated hMSCs was studied via RT‒PCR to examine the effects of different treatments (CBD, THC, HSO) on fat accumulation genes and brown adipose tissue genes. Compared with those of the control (D-hMSC), fat accumulation and adipogenic gene expression differed significantly. PPARγ mRNA expression was significantly greater (0.573-fold ± 0.057) in the THC group than in the D-hMSC group (*p* value ≤ 0.001). Interestingly, PPARγ mRNA expression was slightly downregulated in the HSO-treated groups but was not significantly changed. Moreover, the expression of CCAAT/enhancer-binding protein alpha (CEBPα) was significantly lower in all the groups than in the control group (D-hMSC). Notably, compared with the control, THC treatment downregulated the expression of CEBPα mRNA by 0.654-fold ± 0.303 (p value ≤ 0.001). Similarly, all the treatments significantly downregulated the expression of fatty acid-binding protein 4 (FABP4) mRNA, and the greatest decrease was observed in the THC-treated group. THC treatment decreased FABP4 mRNA levels 0.636-fold ± 0.012-fold. Moreover, HSO significantly downregulated the expression of these genes by more than 0.569-fold ± 0.066 (p value ≤ 0.001). Similarly, perilipin-1 (PLIN-1) mRNA expression was significantly downregulated by at least 31% in all the treatment groups.

**Fig. 2:**
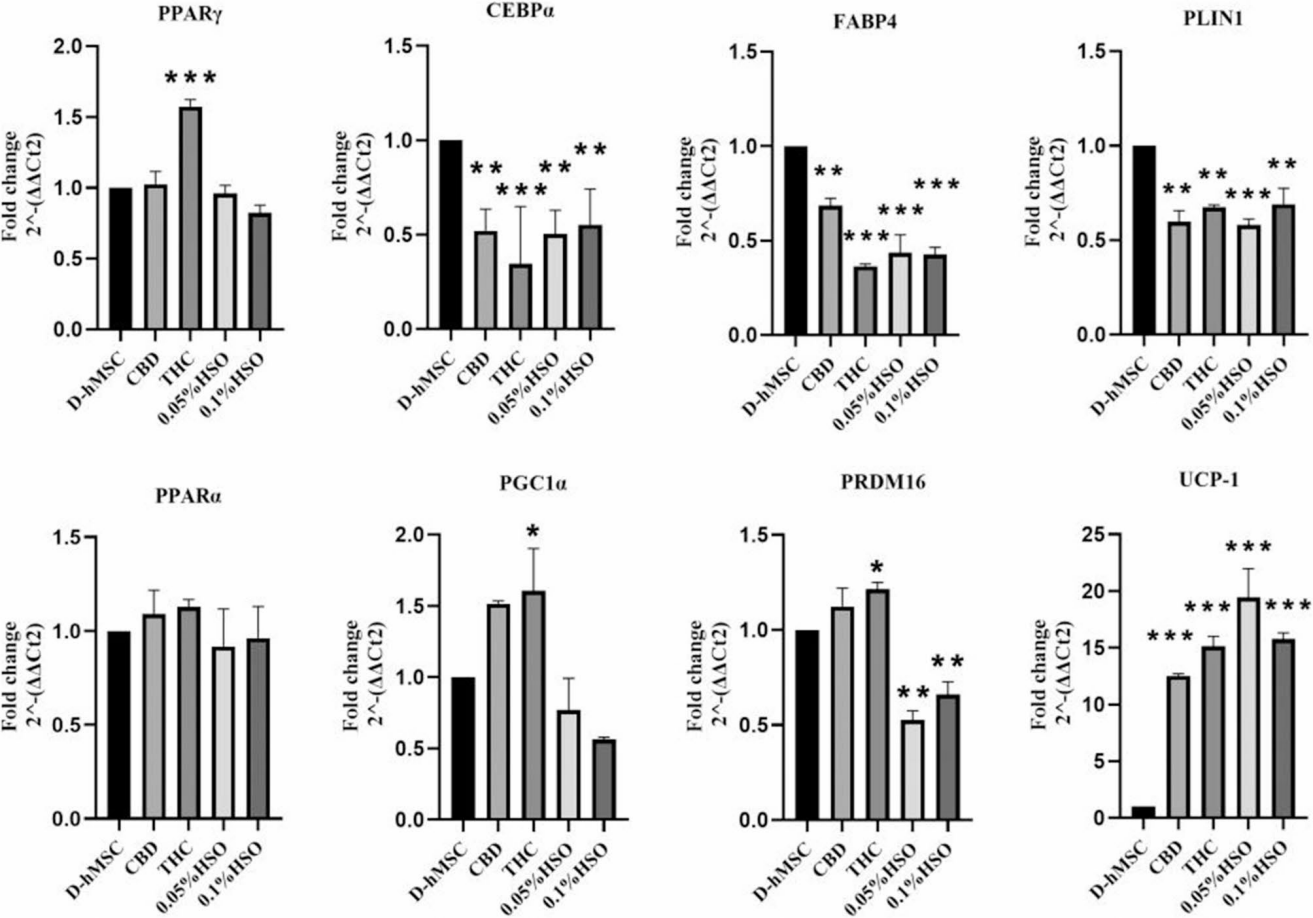
Gene expression. Adipogenic gene (PPARy, CEBPa, FABP4, and PLIN1) and brown adipose gene (PPARa, PGC1a, PRDM16, and UCP1) mRNA levels were measured using quantitative RT-PCR after 14 days. First 72h, cells were cultured in adipose differentiation media; then, 72h of culturing with treatments (CBD, THC, 0.05% HSO, or 0.1% HSO), and finally, maintenance media until day 14th. The biological experiments data presented as mean and standard deviation (SD) of quadruplicate technical replicates (*n*=4) were used to represent all data (mean ± SD), are shown; ANOVA as *p ≤0.05, ** *P*≤0.01, *** *P* ≤0.001

Brown adipose tissue (BAT) gene expression was substantially different from that in the control group (D-hMSC). Compared with that in the control group, peroxisome proliferator-activated receptor-alpha (PPARα) mRNA expression did not differ significantly among the treatment groups. However, it was slightly downregulated in the HSO-treated groups and upregulated in the CBD and THC groups, as shown in Fig. 2. Similarly, HSO treatment decreased the expression of peroxisome proliferator-activated receptor gamma coactivator 1-alpha (PGC1α) and positive regulatory domain zinc finger region protein-16 (PRDM16). Moreover, HSO (0.05% HSO & 0.1% HSO) significantly increased the expression of uncoupling protein-1 (UCP1) (p value ≤ 0.001) by 19.42-fold ± 2.56 and 15.78-fold ± 0.53, respectively. Interestingly, CBD and THC, but not CBD, increased the expression of PGC1α and PRDM16, but these effects were significant only among the THC group. Similarly, CBD and THC significantly increased the expression of UCP1 (p value ≤ 0.001) by 12.52 ± 0.19-fold and 15.11 ± 0.88-fold, respectively.

### ECS receptors and enzymes are regulated by HSO treatment

**Fig. 3:**
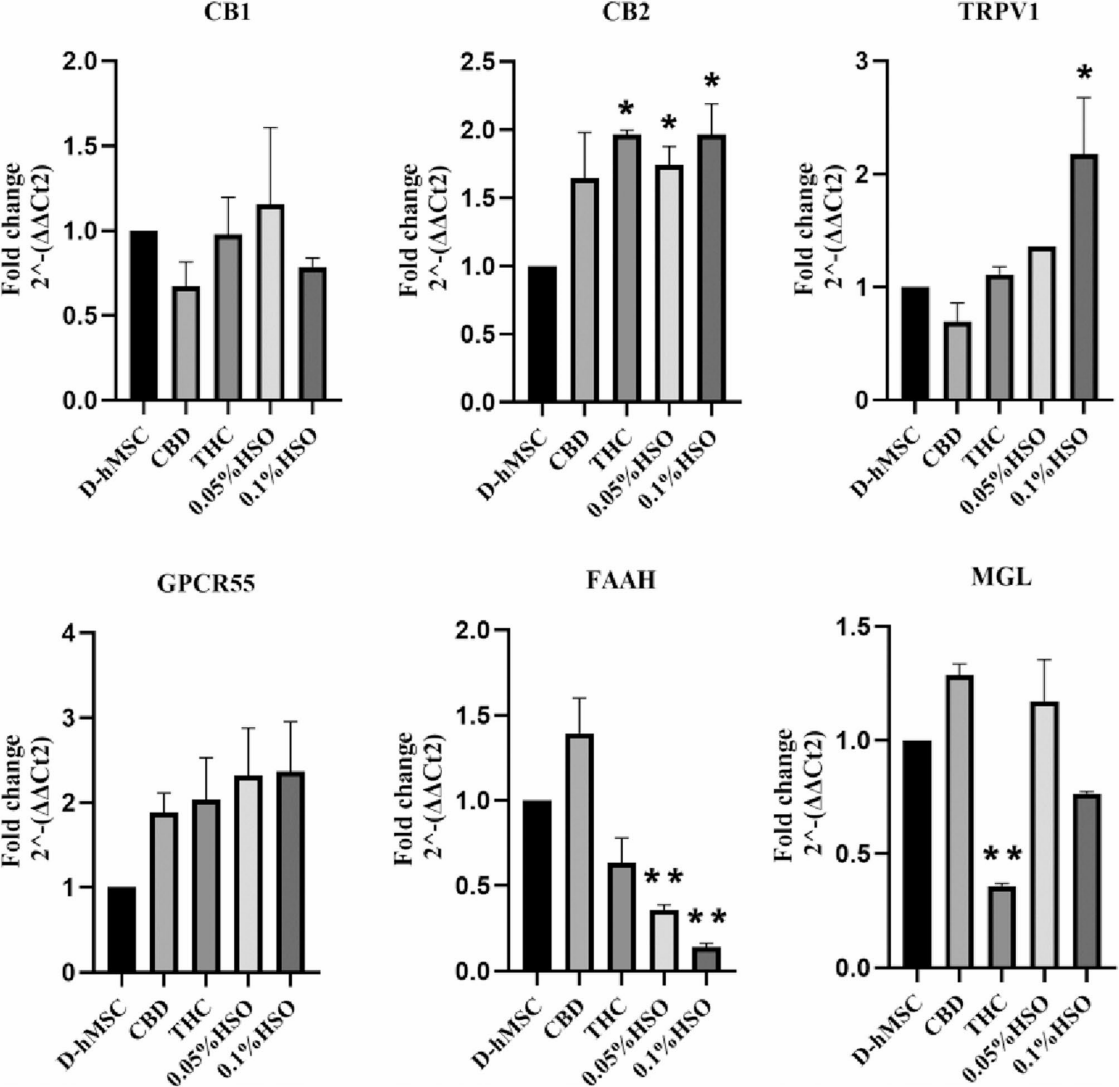
Gene expression. Quantitative RT-PCR was used to measure mRNA levels of endocannabinoid system receptors and enzymes (CB1, CB2, TRPV1, GPCR55, FAAH, and MGL) after culturing in adipose differentiation media for 72h; then, 72h with treatments (CBD, THC, 0.05% HSO, or 0.1% HSO), and finally, maintenance media until day 14th. The biological experiments data presented as mean and standard deviation (SD) of quadruplicate technical replicates (*n*=4) were used to represent all data (mean ± SD), are shown; ANOVA as *p ≤0.05, ** *P* ≤0.01, *** *P*≤0.001

**Fig. 4:**
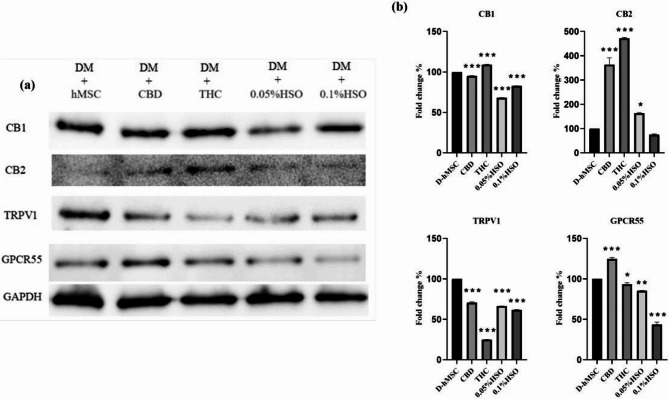
Western blotting. **a** CB1, CB2, TRPV1, and GPCR55 after 72h of adipose differentiation, 72h of CBD, THC, 0.05%HSO, or 0.1%HSO treatment, and maintenance medium until day 14. **b** CB1, CB2, TRPV1, and GPCR55 densitometry. The biological experiments data presented as mean and standard deviation (SD) of quadruplicate technical replicates (*n*=4) were used to represent all data (mean ± SD), are shown; ANOVA as *p≤0.05, ** *P* ≤0.01, *** *P*≤0.001

As HSO and other treatments (CBD and THC) affect adipose tissue differentiation, we wanted to explore the role of the endocannabinoid system, including its receptor and degrading enzyme mRNAs (Fig. 3) and receptor protein expression (Fig. 4), in fully adipogenic differentiated hMSCs. To this end, qRT‒PCR and immunoblotting were performed. The qRT‒PCR results in Fig. 3 show that CB1 mRNA was downregulated in all treatment groups except for 0.05% HSO compared with the control (D-hMSC). Moreover, CB2 mRNA was overexpressed in all the treatment groups by more than 1.6-fold but was significant only in the THC, 0.05% HSO, and 0.1% HSO groups (1.966-fold ± 0.029, 1.741-fold ± 0.136, and 1.966-fold ± 0.224, respectively). Additionally, TRPV1 mRNA expression was increased in all treatment groups except for CBD but was significant only in the 0.1% HSO group, where it was increased by 2.177-fold ± 0.496. Similarly, GPCR55 mRNA was overexpressed more than 2-fold in all treatment groups compared with the control, but the difference was not significant. On the other hand, the groups expressed the fatty acid-degrading enzymes FAAH and MGL differently. The expression of FAAH mRNA was significantly decreased in the HSO groups (0.05% HSO & 0.1% HSO). Moreover, it increased in the CBD group but was not significant. Similarly, CBD treatment resulted in the overexpression of MGL mRNA along with 0.05% HSO. However, MGL mRNA was decreased in the 0.1% HSO and THC groups but was significantly decreased only in the THC group.

Next, western blot quantification via densitometry confirmed the RT‒PCR results (Fig. 4). Densitometric analysis revealed that the CB1 expression level was slightly but significantly lower in all the groups than in the D-hMSC group (p value ≤ 0.001), except for the THC treatment group. Moreover, CB2 expression increased significantly in all the treatment groups except for the 0.1% HSO group. Similarly, TRPV1 and GPCR-55 levels were decreased significantly in all treatment groups except for CBD, which significantly increased the expression of GPCR55 (p value ≤ 0.001). Interestingly, HSO treatment (0.05% HSO and 0.1% HSO) significantly decreased the protein expression of ECS receptors (CB1, TRPV1, and GPCR55).

### HSO regulated inflammatory markers

**Fig. 5:**
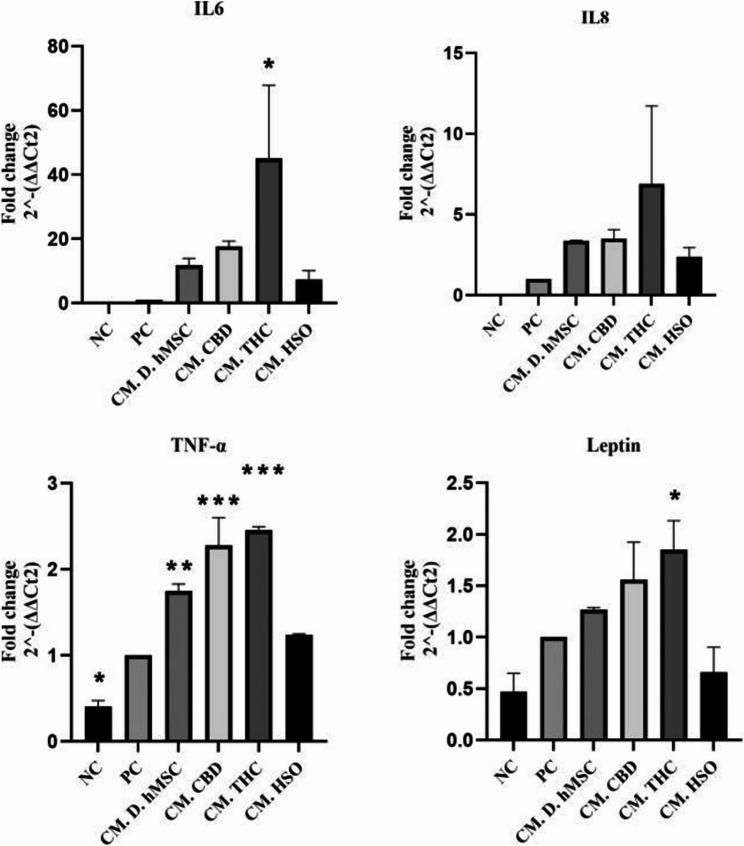
Gene expression mRNA levels of inflammatory markers (IL-6, IL-8, TNF-a, and leptin) of THP-1 cells after 24h incubation with 100ng/ml of Lipopolysaccharide in the condition media collected from adipose differentiated hMSC and treated adipose differentiated hMSC (CBD, THC, HSO) after 14 days. The biological experiments data presented as mean and standard deviation (SD) of quadruplicate technical replicates (*n*=4) were used to represent all data (mean ± SD), are shown; ANOVA as *p ≤0.05, **P≤0.01, *** *P*≤0.001

Given that adipose tissue inflammation is linked to many diseases, we wanted to examine whether treated conditioned media (CM-D-hMSC, CM-CBD, CM-THC, CM-HSO) collected from differentiated hMSCs (14 days) influence inflammation markers. To this end, THP-1 cells were cultured until they reached 70% confluency, after which the medium was replaced with CM for two hours, followed by a 24 h incubation with 100 ng/ml LPS. The results revealed that all the inflammatory mRNAs were overexpressed in all the treatment groups compared with the positive control group (THP-1 + LPS only), except for the leptin mRNA in CM-HSO, which decreased but not significantly (Fig. 5). However, CM-HSO slightly decreased compared with that in CM-hMSCs treatment groups. Furthermore, D-THC increased the expression of all inflammatory genes (IL-6, IL-8, TNF-α, and leptin) by 129.1-fold ± 7.24, 131.6-fold ± 51.31, 2.455-fold ± 0.04, and 1.846-fold ± 0.22, respectively. Overall, In THP-1 macrophage, CM-HSO treatment decreased the expression of IL-6, IL-8, TNF-α, and leptin mRNAs significantly when compared to CBD and THC, respectively.

## Discussion

In this study, we aimed to assess the effects of HSO treatment on adipocyte hyperplasia and preadipocyte to adipocyte maturation, the involvement of the ECS during this process, and the expression of inflammatory markers compared with those of CBD and THC. We found that HSO treated with preadipocytes regulate toward brown-like adipocyte maturation in terms of morphology observed in oil Red O’ and gene expression. The HSO-treated cells were found with spindle and linear shape morphology when compared CBD-treated and THC-treated cells (Fig. 1(a)). In addition, HSO-treated cells contained fewer intracellular lipids in a dose-dependent manner than did all the other treatments (Fig. 1[b]).

The availability of omega 3 and omega 6 fatty acids in cold pressed HSO (Almousa et al. 2024), along with CBD and THC might modulate the expression of CB2 receptor in maturing adipocytes. Prominently, gene expression analysis confirmed that HSO treated adipocytes significant increased mRNA expressions of CB2, GPCR55, UCP-1 and decreased expression of CB1. In this context, Yeliseev et al. (2021) confirmed that cholesterol components increase the basal activation of the CB2 receptor, then alters the pharmacological categorization of these ligands in adipocytes. Increased CB2 represents decreased lipogenesis, stimulation of browning effect and mitochondrial efficiency, rather decreased CB1 represent to increased lipogenesis and hinder mitochondrial efficiency (Yeliseev et al. 2021; Deveaux et al. 2009). The variation in those mRNA expressions were represent the capacity of fatty acid oxidation in adipocytes, which corresponding to the reduced intracellular lipid content in Nile red staining images in HSO treated maturing adipocytes (Fig. 1).

Furthermore, HSO treatment decreased the expression of all fat accumulation genes (PPARγ, CEBPα, FABP4, and PLIN-1) and all BAT genes (PPARα, PGC1α, and PRDM16), with the exception of UCP-1, which was significantly increased (Fig. 2) and leans toward brown-like adipocytes (beige adipocytes); these results align with the characteristics of beige adipocytes (Lizcano 2019; van Eenige et al. 2018). They concluded that beige adipocytes are characterized by a rapid increase in UCP1 in response to stimulation while maintaining low expression of BAT genes such as PGC1α and UCP1 in the absence of stimuli. Interestingly, CBD and THC decreased the expression of almost all lipogenesis- and fat accumulation-related genes (CEBPα, FABP4, and PLIN-1) and increased the expression of all BAT genes (PPARα, PGC1α, PRDM16, and UCP1), which lean toward brown adipocytes. This might be due to the elevated expression of CB2 in these treatment groups.

With respect to the involvement of the ECS in the adipocyte maturation of hMSCs, we studied the gene and protein expression levels of ECS receptors, including CB1, CB2, TRPV1, and GPCR55, along with the gene expression of the ECS-degrading enzymes FAAH and MGL. Our data revealed that a relatively high concentration of HSO downregulated CB1, FAAH, and MGL mRNAs while increasing CB2, TRPV1, and GPCR55 mRNAs. In this context, suppression of CB1 and stimulation of CB2 causes white adipocytes to transform into brown-like fat cells (Harms and Seale 2013; Rossi et al. 2016). Furthermore, CB1 knockout led to lean mice (in vivo), and the use of a CB1 antagonist on white adipocyte cells retrieved from mice increased UCP1 transcription (in vitro) (Perwitz et al. 2010). Similarly, upregulation of TRPV1 inhibited adipogenesis in 3T3-L1 preadipocytes, and obesity was prevented in mice treated with fid TRPV1 agonists for 120 days compared with TRPV1-knockout mice (Zhang et al. 2007). They also reported that visceral fat from obese humans expressed less TRPV1 than did visceral fat from lean humans. However, at the protein level, HSO treatment significantly decreased the protein expression of ECS receptors (CB1, TRPV1, and GPCR55) but not CB2. Interestingly, CBD and THC significantly increased the protein expression of CB2 by 283% and 374%, respectively. This aligns with the finding that higher expression of CB2 induces brown-like adipocytes by increasing BAT genes (Rossi et al. 2016), which is the case with our CBD and THC treatments, which resulted in elevated levels of CB2 mRNA and protein, leading to higher levels of all BAT genes.

With respect to the role of HSO treatment in the expression of inflammatory markers (IL-6, IL-8, TNF-α, and leptin) in THP-1 cells, HSO decreased the expression of all inflammatory marker mRNAs except leptin mRNA; however, HSO had a minimal decrease compared with the CM-D-hMSCs, THC and CBD treatments, respectively (Fig. 5). In contrast, another study revealed that the total phenylpropanamides extracted from hempseed (at a low dose) significantly decreased the brain IL-6 and TNF-α levels in LPS-induced model mice, whereas a high dose of phenylpropanamides might induce toxicity, counteracting the anti-inflammatory state of these compounds. We also noted that CM-THC increased the expression of all inflammatory marker mRNAs by a maximum of 131-fold for IL-8 mRNA and a minimum of 1.8-fold for leptin mRNA.

Miller et al. (2020) shed a light on the effects of THC on inflammation and immune-modulatory function of THC compared with CBD. Nevertheless, most of the in vitro studies tested pure THC compounds alone or in conjunction with CBD (marijuana) and on different cell types than hMSCs. They also noted a critical and fatal point, which is the lack of concentration at which THC exerts its anti-inflammatory effects. This could be the case in our study, where administering a large amount of THC negated its ability to suppress the production of inflammatory cytokines.

Another possibility is the expression levels of cannabinoid receptors in THP-1 cells. Ihenetu et al. (2003) reported that the type of cell and type of inflammatory stimulus used may influence cannabinoid receptors differently and consequently activate either pro- or anti-inflammatory responses. Similarly, Han et al. (2009) reported that CB1 activation in human macrophages leads to a proinflammatory state, whereas CB2 activation results in an anti-inflammatory state. In addition, they reported that human macrophages exhibit significant overexpression of CB1 receptors in response to inflammatory and atherogenic stimuli. This might be the cause of our present data, where the use of LPS on THP-1 cells activated the CB1 receptor, and as THC has a strong affinity for CB1, it led to a proinflammatory state.

## Conclusion

Overall, the availability of balanced ratios of omega 3/omega 6 PUFAs and CBD in HSO favors in maintaining optimal ECS ligands in adipocytes. Our current study revealed that HSO treatment might promote the maturation of hMSC preadipocytes toward brown-like adipose tissue, which evident morphologically. ECS might mediate this effect, as HSO treatment downregulates the CB1 receptor and increases the CB2 receptor at the mRNA and protein levels. In addition, HSO treatment decreased inflammatory marker of IL-6, IL-8, TNF-α, and leptin compared to untreated cells; however, HSO treatment resulted in a minimalized the provoking of inflammatory cytokines compared with CBD and THC treatments in THP-1 cells. In conclusion, the potential of HSO in promoting the development of brown fat characteristics through the ECS and its effect on inflammation status offers an intriguing area for future research and therapeutic interventions.

## Supplementary Information

Supplementary Material 1.

Supplementary Material 2.

## Data Availability

The data will be made available upon request.
