# Supplementary material for: Adipogenicity-induced human mesenchymal stem cells treated with hemp seed oil stimulate brown-like adipocytes and decrease adipokine levels through the activation of cannabinoid receptor 2 (CB2)
Source: J Cannabis Res. 2025 Nov 19;7:95. doi: 10.1186/s42238-025-00343-2 (PMC12628899; doi:10.1186/s42238-025-00343-2)

Supplementary file: Uncropped Gels and Blots image(s)

1. CB1 - Entire gel and Blot

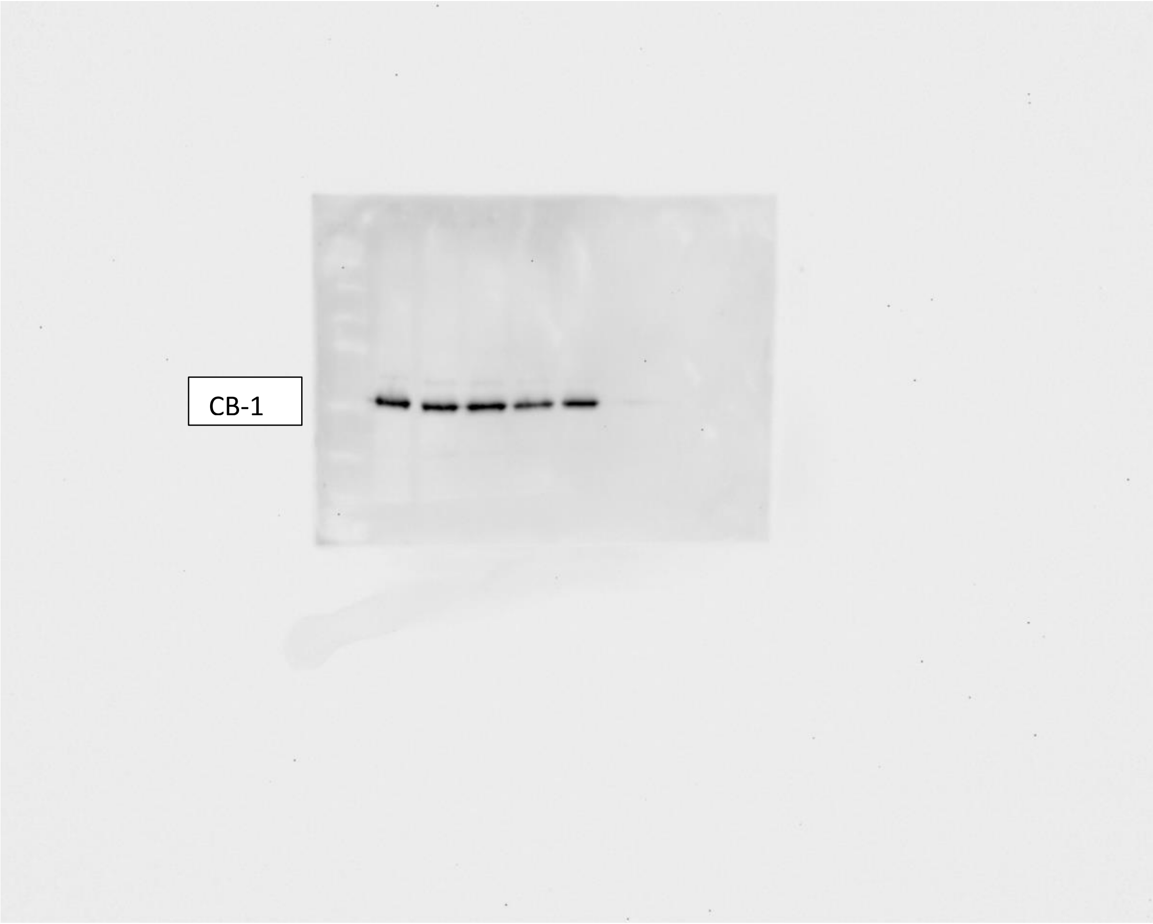

2. CB-2 lower kDa- Entire gel and Blot

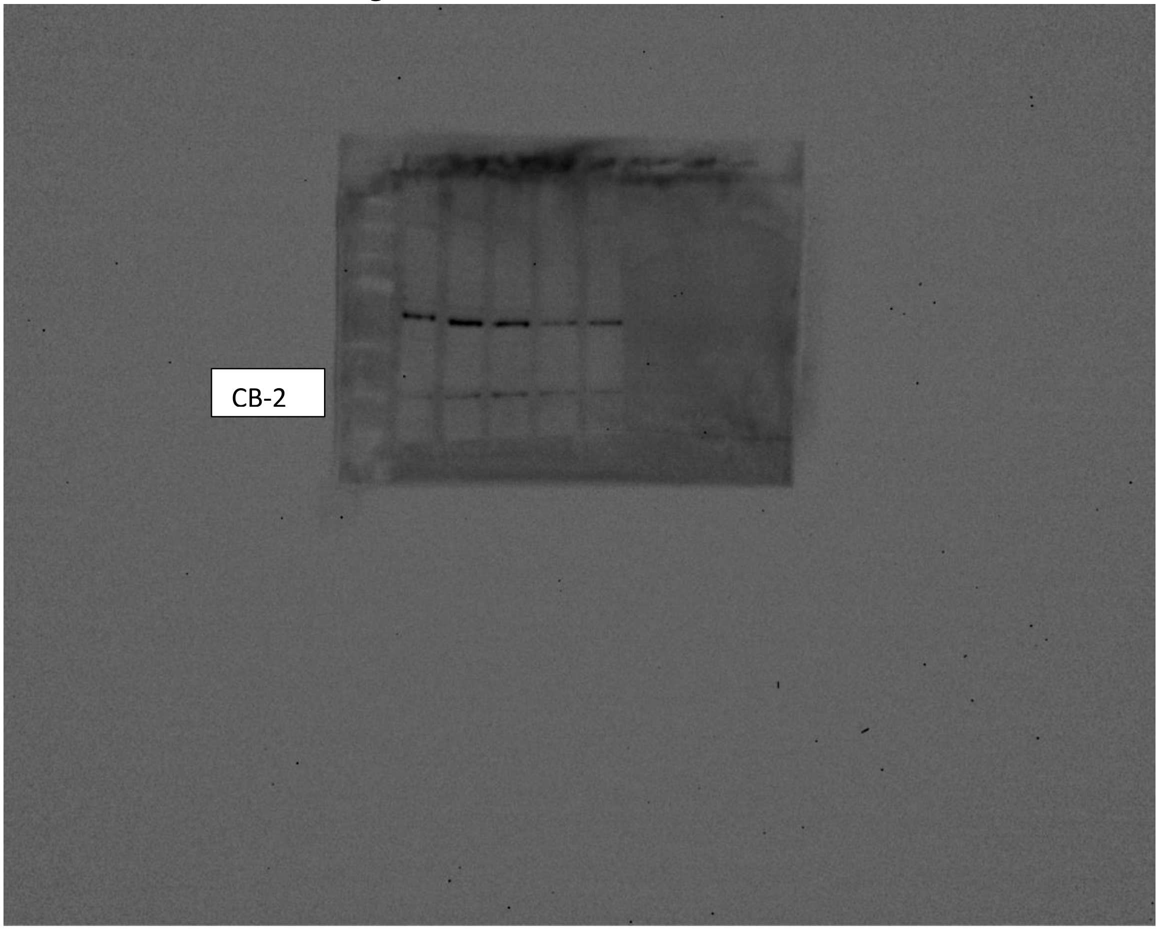

3. TRPV1 - Entire gel and Blot

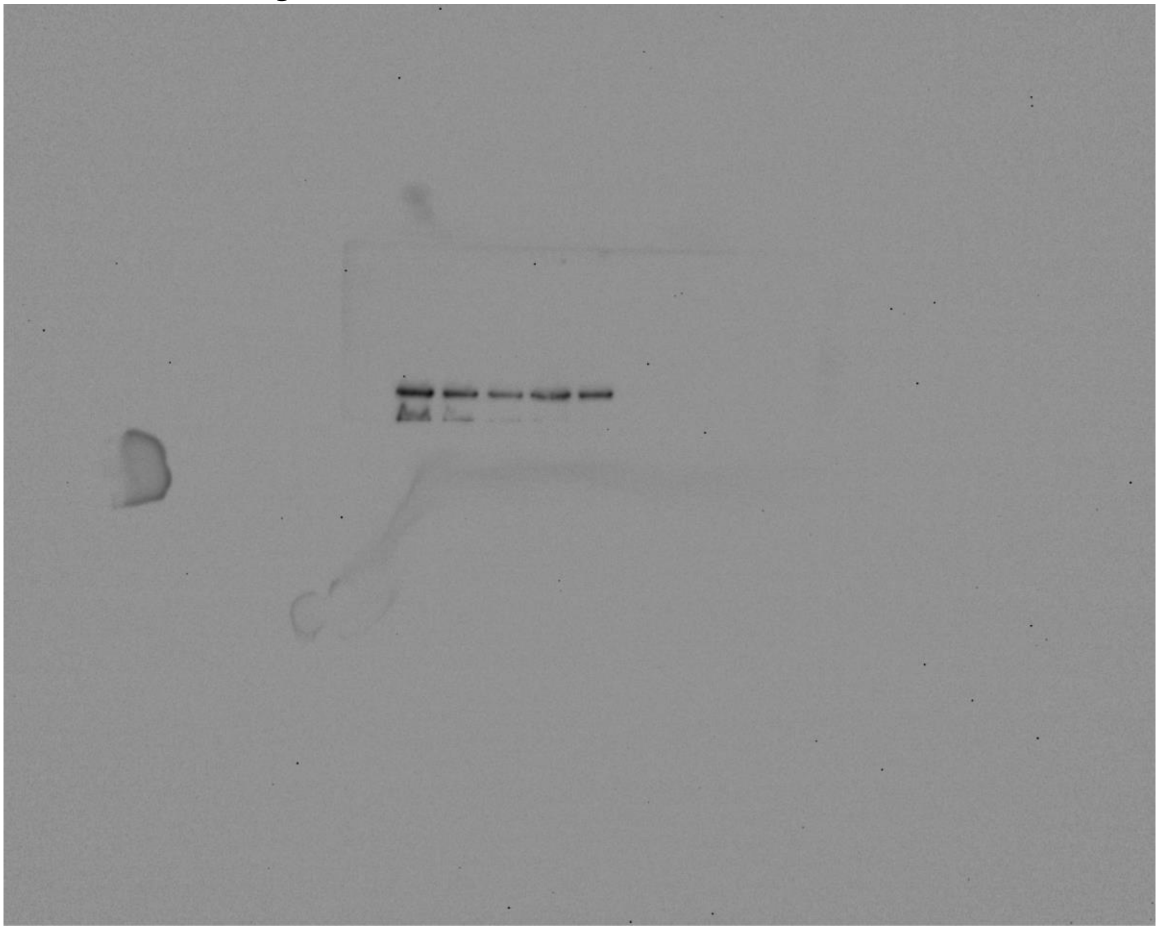

4. GPCR55 - Entire gel and Blot

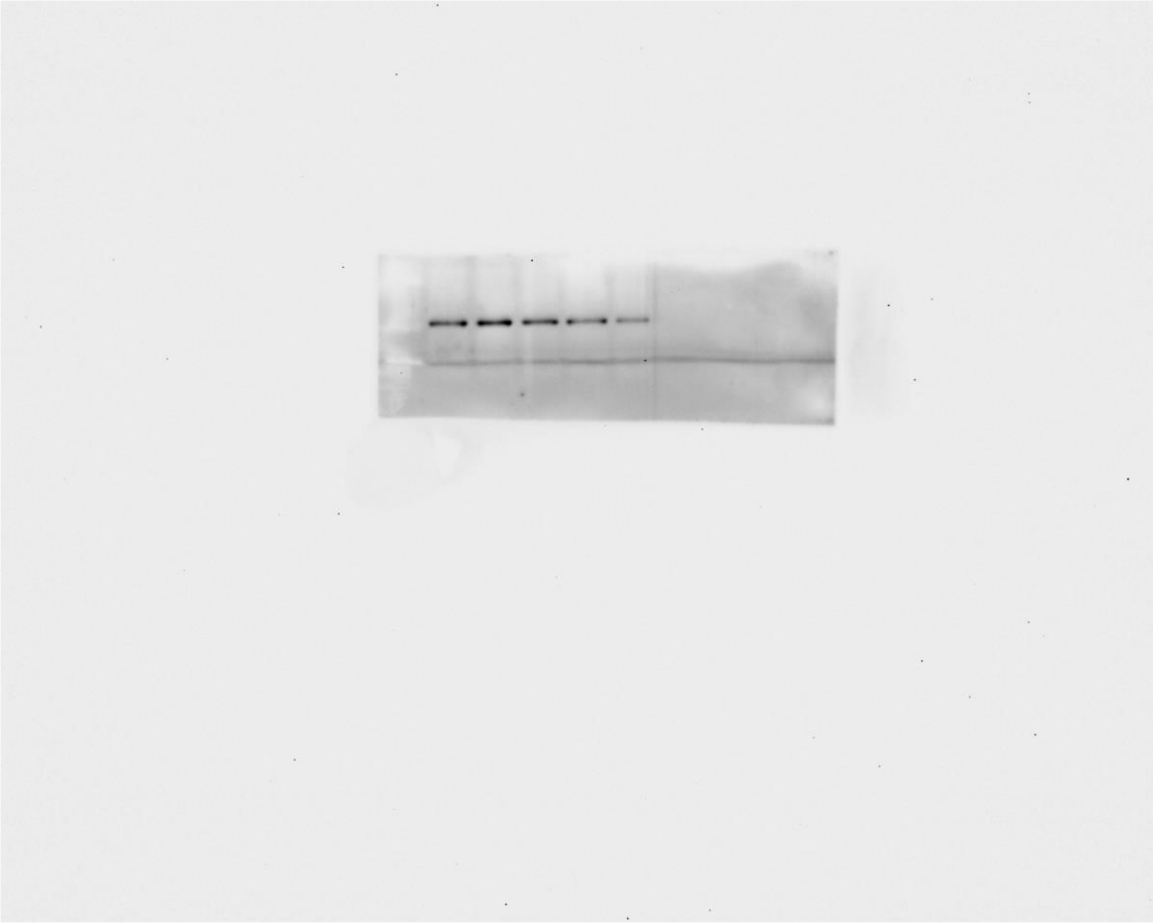

5. GAPDH- Entire gel and Blot

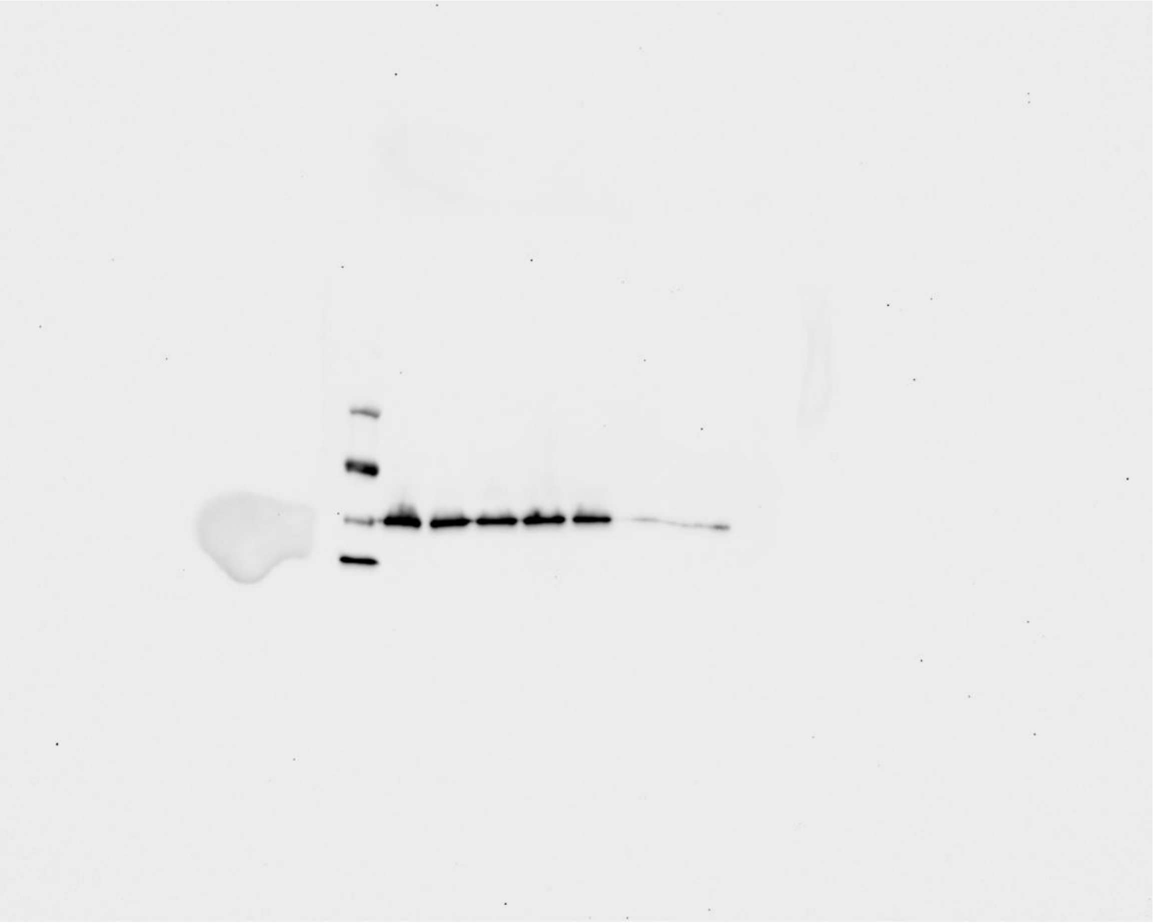

Supplement: Supplementary file 1 — Supplementary Material 1. [file 42238_2025_343_MOESM1_ESM.pdf]
