# Supplementary material for: Adipogenicity-induced human mesenchymal stem cells treated with hemp seed oil stimulate brown-like adipocytes and decrease adipokine levels through the activation of cannabinoid receptor 2 (CB2)
Source: J Cannabis Res. 2025 Nov 19;7:95. doi: 10.1186/s42238-025-00343-2 (PMC12628899; doi:10.1186/s42238-025-00343-2)

**Supplementary file: Uncropped Gels and Blots image(s)**

1. CB1 - Entire gel and Blot


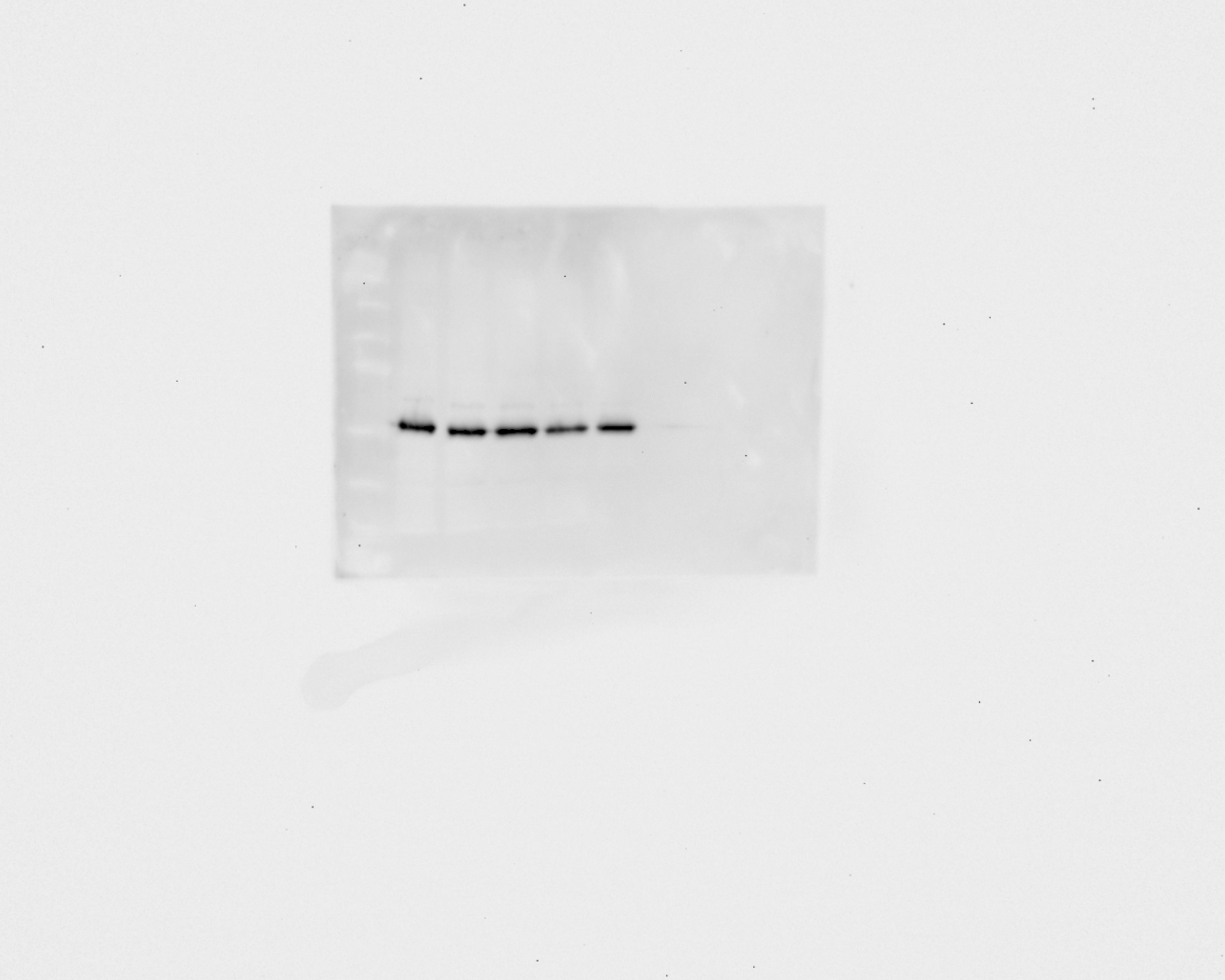


CB-1

1. CB-2 lower kDa- Entire gel and Blot


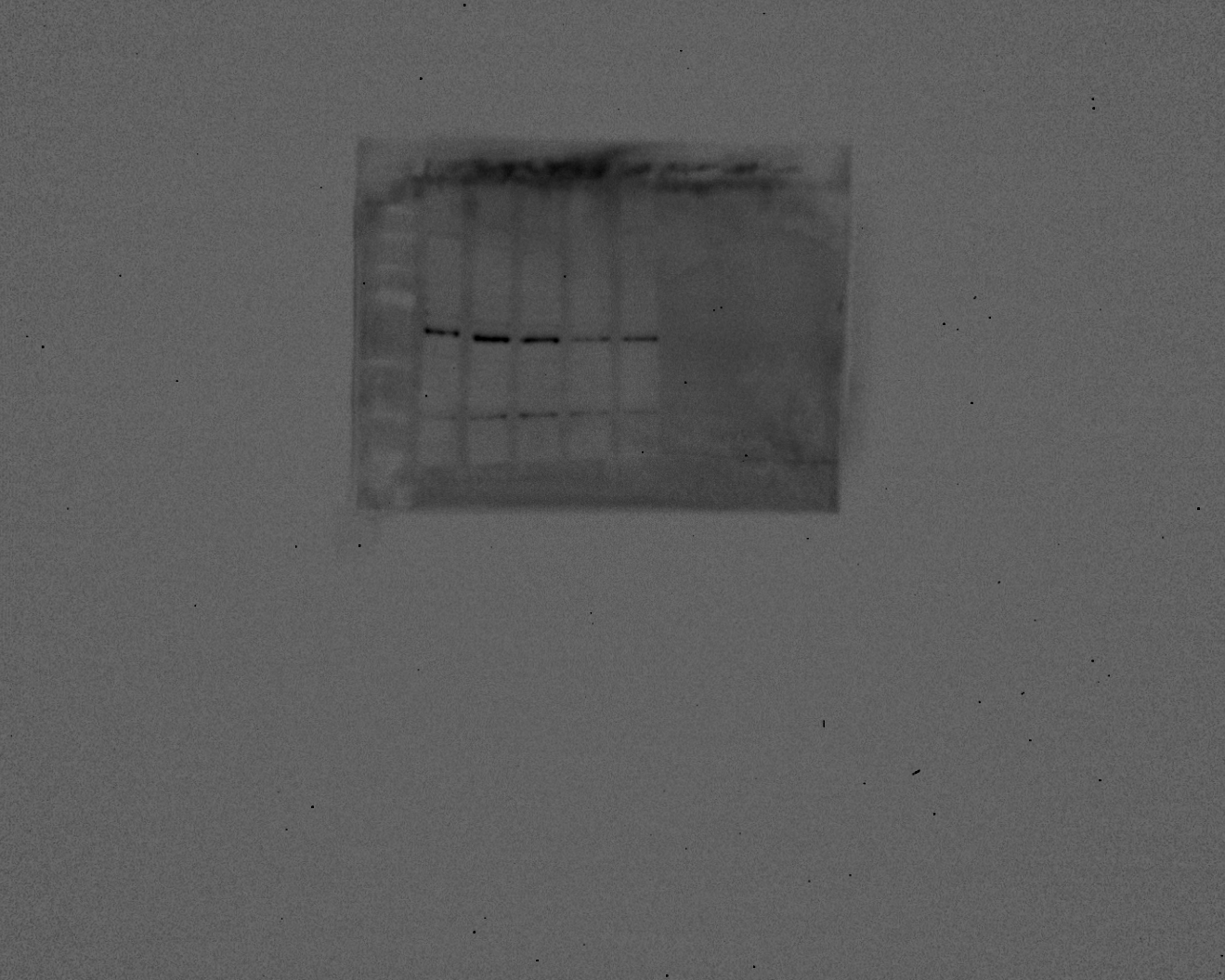


CB-2

1. TRPV1 - Entire gel and Blot


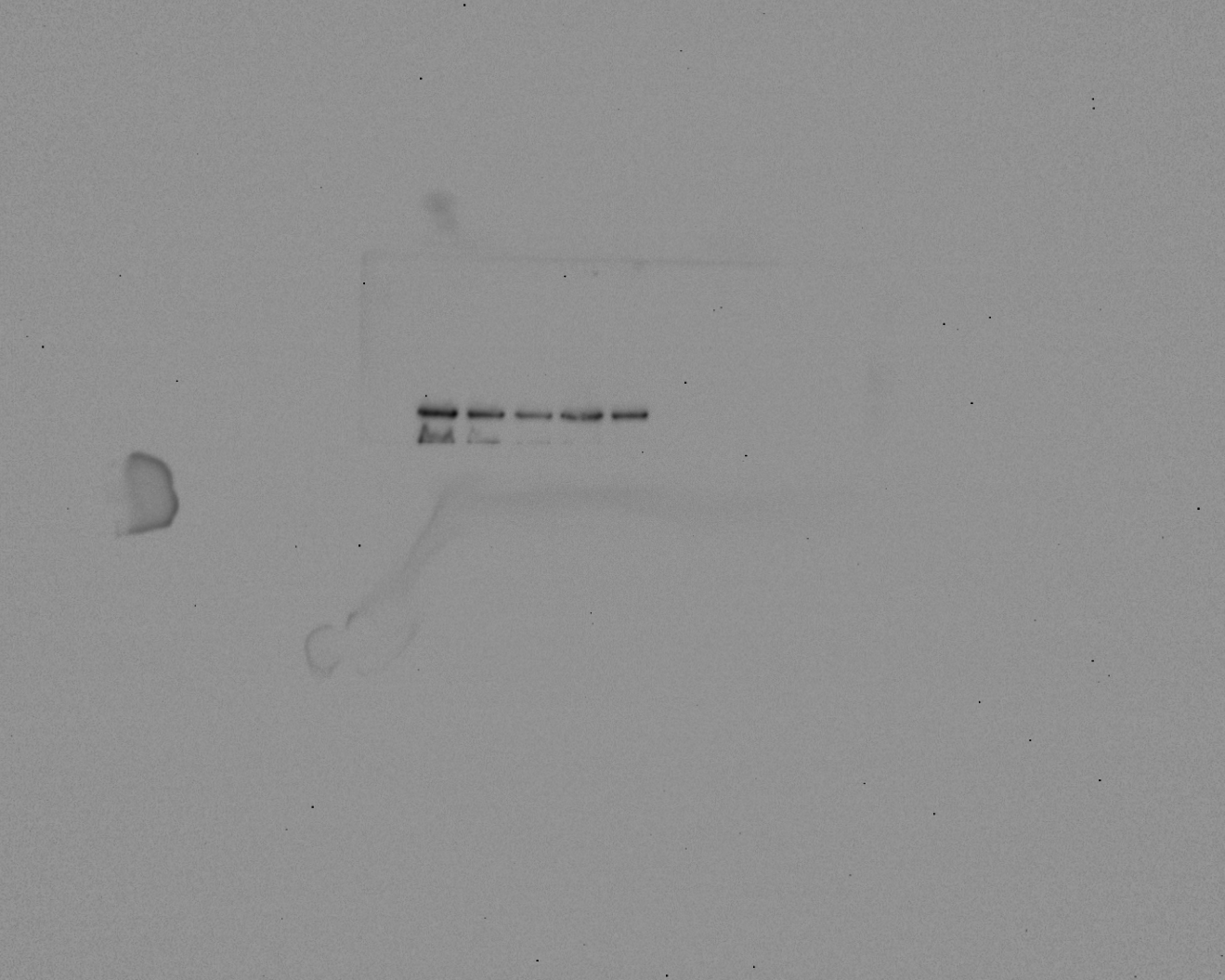


1. GPCR55 - Entire gel and Blot


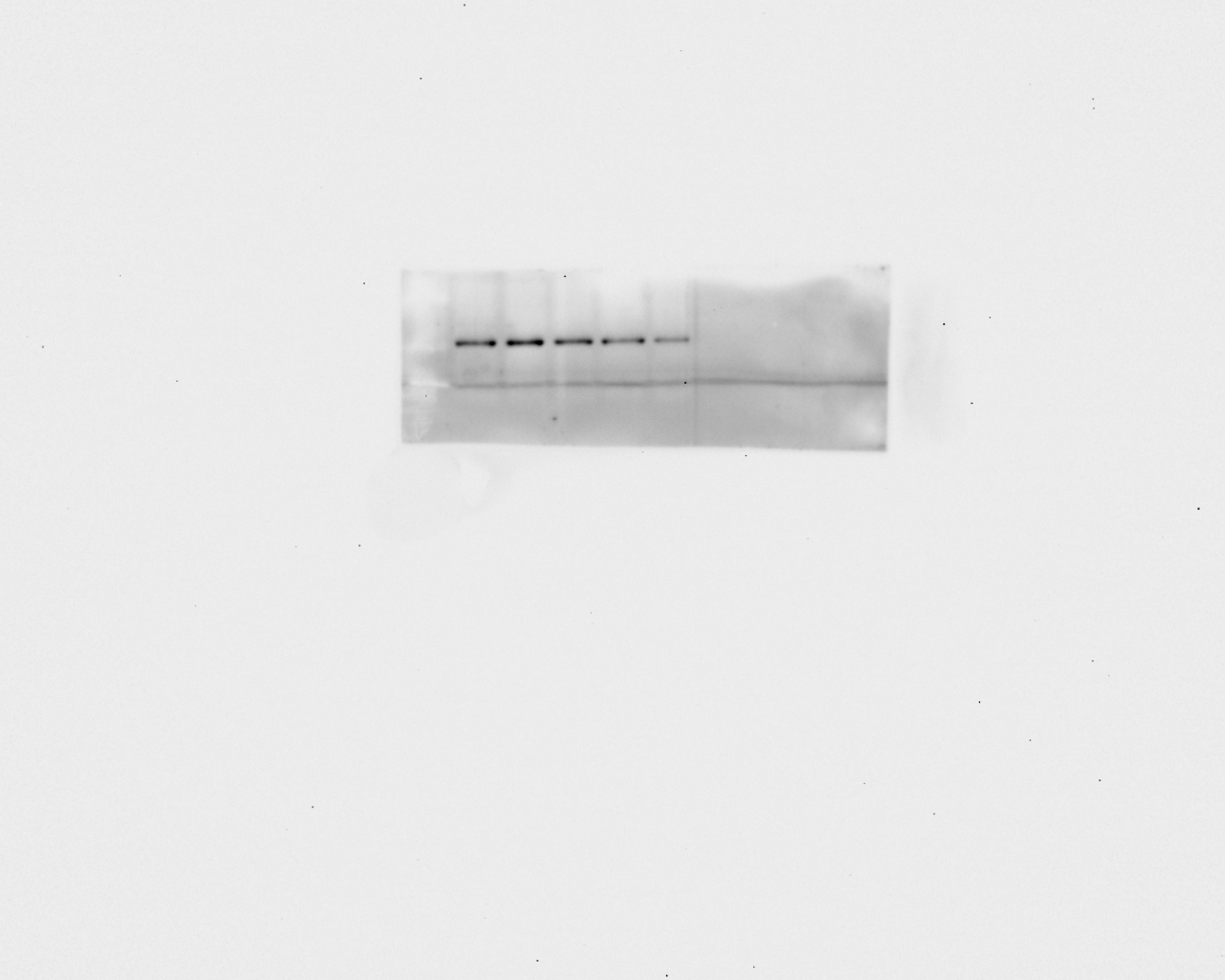


1. GAPDH- Entire gel and Blot


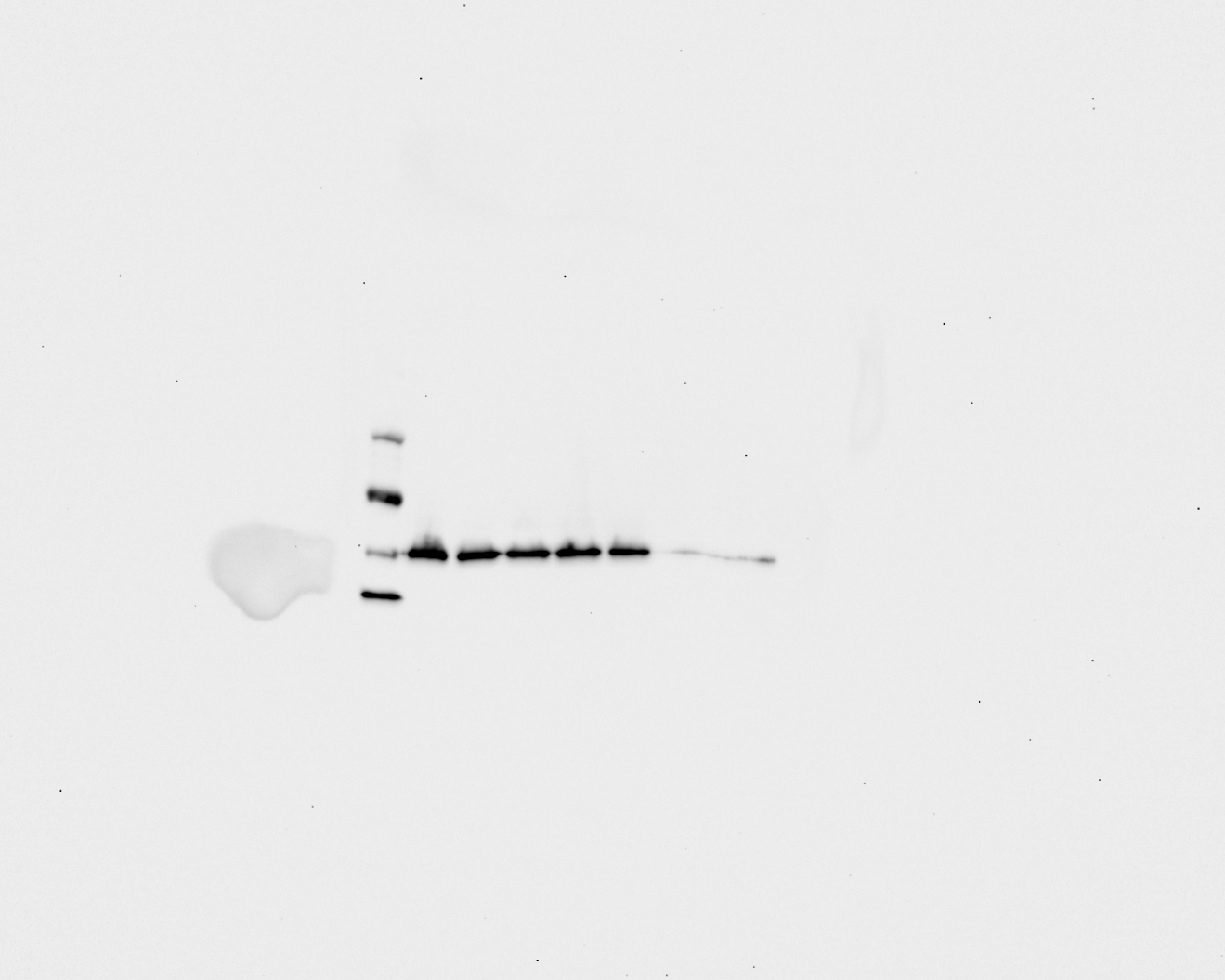

Supplement: Supplementary file 2 — Supplementary Material 2. [file 42238_2025_343_MOESM2_ESM.docx]
